# Engine Failure in Axo-Myelinic Signaling: A Potential Key Player in the Pathogenesis of Multiple Sclerosis

**DOI:** 10.3389/fncel.2021.610295

**Published:** 2021-02-10

**Authors:** Talia Bergaglio, Antonio Luchicchi, Geert J. Schenk

**Affiliations:** Department of Anatomy and Neurosciences, Amsterdam Neuroscience, Amsterdam University Medical Center, Amsterdam MS Center, Amsterdam, Netherlands

**Keywords:** oxidative stress, mitochondria, axo-myelinic synapse, multiple sclerosis, neurodegeneration

## Abstract

Multiple Sclerosis (MS) is a complex and chronic disease of the central nervous system (CNS), characterized by both degenerative and inflammatory processes leading to axonal damage, demyelination, and neuronal loss. In the last decade, the traditional *outside-in* standpoint on MS pathogenesis, which identifies a primary autoimmune inflammatory etiology, has been challenged by a complementary *inside-out* theory. By focusing on the degenerative processes of MS, the axo-myelinic system may reveal new insights into the disease triggering mechanisms. Oxidative stress (OS) has been widely described as one of the means driving tissue injury in neurodegenerative disorders, including MS. Axonal mitochondria constitute the main energy source for electrically active axons and neurons and are largely vulnerable to oxidative injury. Consequently, axonal mitochondrial dysfunction might impair efficient axo-glial communication, which could, in turn, affect axonal integrity and the maintenance of axonal, neuronal, and synaptic signaling. In this review article, we argue that OS-derived mitochondrial impairment may underline the dysfunctional relationship between axons and their supportive glia cells, specifically oligodendrocytes and that this mechanism is implicated in the development of a primary cytodegeneration and a secondary pro-inflammatory response (*inside-out*), which in turn, together with a variably primed host’s immune system, may lead to the onset of MS and its different subtypes.

## Introduction

Multiple Sclerosis (MS) is a complex, chronic progressive disorder of the central nervous system (CNS) and the most prominent cause of neurological disability in young adults (Noseworthy et al., 2000; Compston and Coles, 2002; Stys et al., 2012). Comprising of damage to both white and gray matter regions of the brain, evidence from histopathological findings identifies demyelination and axonal damage, as well as microglia activation, synaptic and neuronal loss as hallmarks of the disease (Geurts and Barkhof, 2008; Brinar and Braun, 2013; Klaver et al., 2013; Calabrese et al., 2015). Conflicting ideas have been proposed to explain the etiology of MS, particularly the origin of lesion formation and disease progression. The indisputable involvement of inflammatory processes has resulted in the development of the main model of MS, namely *outside-in*, whereby a dysregulated immune system in the periphery attacks elements within the CNS, causing demyelination and tissue damage (Stys et al., 2012; Dendrou et al., 2015). Once inflammation becomes compartmentalized within the CNS and compensatory mechanisms can no longer overcome the chronic inflammatory processes, the progressive phenotype takes over and current anti-inflammatory therapeutic strategies are no longer effective (Lassmann et al., 2012). Alternatively, MS may originate from the “*inside-out*,” with a primary degenerative episode, possibly evolving around the axo-myelinic synapse (AMS), disrupting the dynamic communication between axons and their myelinating oligodendrocyte, thus initiating a secondary inflammatory response due to the highly antigenic debris derived from myelin breakdown (Trapp and Nave, 2008; Stys et al., 2012). Consequently, the strength of the convolution between degenerative and inflammatory processes will derive the clinical course of the disease.

According to the *inside-out* model, more focus should be aimed at understanding the mechanisms underlying the neurodegenerative processes of MS. Observations of early-stage lesion formation, in both experimental models and human MS, corroborate on the presence of axonal injury in still myelinated axons, suggesting that, at least in some cases, degenerative CNS pathology precedes the peripheral inflammatory attack (Brück and Stadelmann, 2003; Barnett et al., 2009; Edgar et al., 2010; Nikić et al., 2011; Lubetzki and Stankoff, 2014). A potential mechanism driving *inside-out* immunopathogenesis may evolve around the myelinating unit (Stys, 2013). Here, earlier studies in mice revealed a crucial role of myelin molecules and oligodendroglial support in the maintenance of axonal integrity (Griffiths et al., 1998; Yin et al., 1998; Lappe-Siefke et al., 2003). More specifically, axonal metabolic support requires specific axo-myelinic communication, and disruptions to this relationship may induce primary axonal injury and potentially long-lasting myelin abnormalities (Micu et al., 2016).

Axonal and oligodendroglial processes are highly energy-consuming and as such, are vulnerable to metabolic challenges (Micu et al., 2018). Due to the crucial functions mitochondria play as the power plants of a cell, it is not surprising that both genetic and environmental factors altering their functions have profound downstream effects on axonal health (Campbell et al., 2014; Witte et al., 2014). One of the mechanisms that cause injury to mitochondria is Oxidative stress (OS), defined as an imbalance in Reactive Oxygen Species (ROS) production against the cell’s antioxidant defenses (Betteridge, 2000). Despite the established involvement of ROS-mediated tissue injury in MS inflammatory processes, the induction of pro-apoptotic mechanisms by OS as well as its impact on the mitochondrial respiratory chain may induce a state of energy deprivation that, if chronic, may ultimately initiate a cascade of degenerative processes, including axonal and neuronal death (Lassmann and van Horssen, 2011; Franklin et al., 2012; Stys, 2013; Ohl et al., 2016).

This mini review article aims to highlight a specific component of MS pathogenesis by lending credence to the role of OS-derived mitochondrial dysfunction as a potential key mechanism contributing to an unstable AMS, which may ultimately contribute to a primary cytodegeneration in MS pathogenesis. To do so, evidence supporting an *inside-out* view of MS will be presented, together with the recent insights into the function of axo-myelinic neurotransmission and the role of axonal mitochondria and OS-associated mitochondrial dysfunction. Here, we propose that OS-derived mitochondrial impairment may underline the dysfunctional relationship between axons and their supportive glia cells, which later initiates the primary cytodegeneration and secondary inflammatory processes of MS.

## Multiple Sclerosis as A Primary Cytodegenerative Disease

Although an *outside-in* view of MS pathogenesis cannot be disregarded, the *inside-out* model is equally plausible and equally consistent with the majority of pathological observations. When viewing MS pathogenesis from the *inside-out*, axonal injury and myelin defects likely act as the initiators of the degenerative processes underlying the disease, due to their observation even in the absence of inflammation in human brain samples (Trapp et al., 1998; Traka et al., 2016). Histopathological analysis of patient material from very early stages of the disease has revealed myelin abnormalities, specifically involving the inner myelin sheath and oligodendrocyte loss with little peripheral inflammatory infiltration but with a more pronounced innate immune response (Rodriguez and Scheithauer, 1994; Aboul-Enein et al., 2003; Barnett and Prineas, 2004; Henderson et al., 2009). Moreover, the ineffectiveness of available anti-inflammatory treatments in halting disease progression further exacerbates the presence of underlying cytodegenerative processes driving MS progression (Seewann et al., 2009; Hawker, 2011; Lassmann, 2013). This evidence is corroborated by genetic studies. Immunologically relevant genes are significantly overrepresented in genome-wide association studies (Compston and Coles, 2002). Sawcer et al. (2011) found a strong correlation with immune-related genes within patients with RRMS mainly, which is to be expected due to the great inflammatory character of this MS variant and may explain symptomatic heterogeneity due to a variably primed host’s immune system (Stys et al., 2012). Interestingly, however, when a subgroup analysis of only PPMS patients was performed, no associations with genes related to the immune system were found, further indicating that these immune-related factors may only determine the intensity of autoimmune response to a degenerative brain (Stys and Tsutsui, 2019). Instead, a general state of chronic excitotoxicity seems to, at least in part, drive the degenerative processes of MS, whereby variations in genes related to glutamate signaling prevailed in PPMS patients (Baranzini et al., 2010; Strijbis et al., 2013). Due to the chronic and progressive fate of MS, which will rarely be fatal in the early stages, all human neuropathological studies will inevitably mirror a combination of degenerative processes and inflammatory reactions that have evolved over many months or years (Stys, 2013). Hence, it is crucial to critically acknowledge that a single histopathological snapshot in time may not be fully representative of the initial events of MS pathogenesis.

If we consider progressive MS to reflect the real pathogenic mechanisms of the disease, then the origin of lesion formation lays within episodes of axonal injury and disruptions to axo-myelinic communication (Lassmann et al., 2012; Friese et al., 2014; Mahad et al., 2015; Guttmann et al., 2016). Thus, axonal injury may start at an early, and yet not observable, stage of the disease and it does not initially manifest in neurological disability (Trapp et al., 1998). Indeed, the CNS comprises many reparative mechanisms that allow for the compensation of axonal loss (Bjartmar et al., 2003). Once acute demyelination progresses into a more chronic state, demyelinated axons hardly survive and degenerative mechanisms become more prevalent. Approximately up to 60–70% of axonal loss was estimated in chronic white matter lesions in severely disabled MS patients (Mews et al., 1998). It is still hypothetical which exact mechanism may generate damage to the axons and whether axonal injury may represent a primary degenerative process, or maybe caused by secondary, non-inflammatory, processes (Stys et al., 2012). However, disruption of the close dynamic relationship between axons and their insulating myelin sheaths has been identified as a potential mode of action leading to a state of energy deficiency and, consequently, axonal injury (Tsutsui and Stys, 2013; Simons et al., 2014).

## Axon-Glia Interaction

The architecture of the axo-myelinic unit is very intricate. Although the myelin sheath supports the electrical properties of the axon, it simultaneously limits the access of the axon to its extracellular environment (Nave, 2010; Simons et al., 2014). Nevertheless, the studies cited provide strong evidence for the implication of the myelinating oligodendrocytes in sustaining the axons by providing the necessary metabolic support (Stys, 2011; Fünfschilling et al., 2012; Lee et al., 2012; Micu et al., 2016). The large and complex crosstalk network between axons and oligodendrocytes constitutes an essential part of the proper functioning of the CNS and exposes the system to a diffuse vulnerability to disorders affecting the myelin (Ortiz et al., 2016).

**Figure 1:**
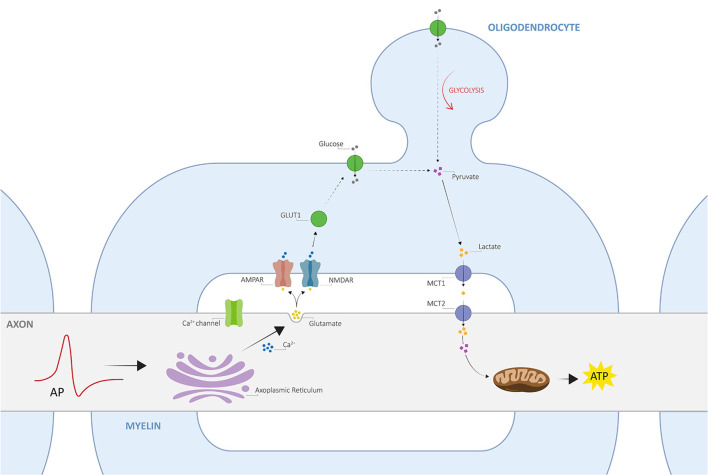
The architecture of axo-myelinic neurotransmission. Traversing action potentials cause depolarization of the axon. The latter is sensed by voltage-gated Ca^2+^ channels, which in turn activate and cause intra-axonal Ca^2+^ release from the axoplasmic reticulum. This stimulates the fusion of glutamatergic vesicles and the consequent release of glutamate into the periaxonal space, which in turn activates myelinic AMPA and NMDA receptors, located on the innermost myelin sheaths (AMPARs and NMDARs, respectively), finally promoting Ca^2+^ influx into the myelin cytoplasm. Myelin receptor activation further results in the recruitment of glucose transporter type 1 (GLUT1), increased uptake of glucose, and the stimulation of glycolysis by the oligodendrocyte, where the production of pyruvate and lactate is enhanced. Pyruvate is then used as an energy supply by myelinic mitochondria, whereas lactate is transported across the periaxonal space and into the axon by monocarboxylate transporters 1 and 2 (MCT1 and MCT2). Finally, lactate is used to fuel aerobic metabolism by axonal mitochondria for the efficient internodal production of ATP. Adapted from Micu et al. (2018); with permission from the authors and SpringerNature.

Despite the close relationship between axons and myelin, only recently, an activity-dependent communication between the two was proposed, namely the axo-myelinic synapse, whose arrangement presents very similar features to the traditional interneuronal synapses (Stys, 2011; Micu et al., 2016, 2018). One critical function of the AMS is thus to couple electrical activity along the axons to the metabolic output from the oligodendrocytes. Remarkably, axons may signal the oligodendrocyte, through AMPA and NMDA myelinic receptors, to supply certain metabolites to fuel the electrically active fiber in response to physiological stimuli; as a result, the glial cells will transfer such metabolites to the internodal axon, providing it with the necessary energy support for signal conductance (Stys, 2011; Micu et al., 2018). In this view, Micu et al. (2016, 2018) propose a potential mechanism of neurotransmission underlying the tightly orchestrated complex directed by the AMS, which is further described in Figure 1.

The continuous supply of energy along the entire length of the myelinated axon is crucial for the efficient conduction of action potentials as well as for the proper maintenance of neuronal functioning, including its myelinating system. Consequently, the energy supply from the myelinating oligodendrocytes is suggested to be of vital importance to fuelling the axon, following transient increases in energy demands and, more generally, to the vast dynamic range of firing frequencies of myelinated axons (Trevisiol et al., 2017). Glucose is the primary energy source in the adult brain and it fuels neuronal activity *via* aerobic respiration by mitochondria (Su et al., 2009). Given the crucial role of mitochondria in driving the majority of cellular processes by providing the necessary energy, it is not surprising that mitochondrial dysfunction can result in significant neuronal injury and degenerative processes (Lin and Beal, 2006; Mahad et al., 2008; Witte et al., 2010).

The balance between ROS production and antioxidant defenses under normal physiological conditions can be disrupted with an overproduction of free radicals, namely OS. Oxidants can play a dual role as both toxic and beneficial compounds, the latter due to their crucial role as essential signaling molecules (Pham-Huy et al., 2008). They are produced from both endogenous (e.g., cell metabolism) and exogenous (e.g., cigarette smoking, environmental toxins, chronic physiological stress, iron overload, inflammation, et cetera) sources and can contribute to disease *via* disruptions to cellular homeostasis by redox signaling (Bhattacharyya et al., 2014; Basria et al., 2019). Over time, exposure to multiple inciting factors, as well as the failure of enzymes responsible for redox homeostasis and detoxification activities, may trigger a chronic imbalance between ROS production and antioxidant defenses (Bhattacharyya et al., 2014; Basria et al., 2019). Excess free radicals can lead to mitochondrial dysfunction by inducing mitochondrial DNA mutations, damage to its respiratory chain, alteration of its membrane permeability, and by influencing Ca^2+^ homeostasis and mitochondrial defense systems (Guo et al., 2013). Once damaged, mitochondrial dysfunctional processes can further amplify OS and generate tissue injury *via* three crucial mechanisms, including energy failure, induction of apoptosis, and enhanced production of ROS (Witte et al., 2014). Energy deficiency derived from mitochondrial dysfunction poses the axon to a state of ‘virtual hypoxia’, whereby axon-glia energy metabolism would directly influence axon-myelin transmission and result in chronic and progressive damage to the axons (Lassmann et al., 2012). More specifically, when energy failure occurs, sodium ions begin to accumulate within the axon, causing, together with membrane depolarization, the reverse mode of action of the Na^+^-Ca^2+^ exchanger (Franklin et al., 2012; Campbell et al., 2014). As a result, detrimental levels of calcium ions build up to a point where they can interfere with axon survival and lead to axonal injury (Tsutsui and Stys, 2013). Through *in vivo* calcium imaging, Witte et al. (2019) have shown that activation of calpains, Ca^2+^-dependent, non-lysosomal proteases, upon increased levels of axoplasmic Ca^2+^, can promote the breakdown of the cytoskeleton as well as oncotic axonal swelling. Activated calpains are also responsible for the permeabilization of lysosomal membranes, which in turn causes the release of the lysosomal hydrolytic enzyme Cathepsin, an important mediator of apoptosis (Czaja, 2001). Both activation of Ca^2+^-dependent proteases, as well as mitochondrial impairment, have been associated with the degradation of myelin-associated glycoprotein (MAG), an adhesive protein located on the periaxonal surface of myelin sheaths and responsible for the inhibition of axon regeneration and the control of myelin formation and maintenance (Kroemer and Jäättelä, 2005; Zong and Thompson, 2006). MAG loss, together with other myelinic proteins, is thought to destabilize the AMS by dysregulating the axon cytoskeleton and by inducing myelin breakdown, further exacerbating myelin-axon communication (Schnaar and Lopez, 2009).

## Mitochondrial Oxidative Stress in Axo-Myelinic Neurotransmission: A Key Player in Ms Primary Degeneration?

**Figure 2:**
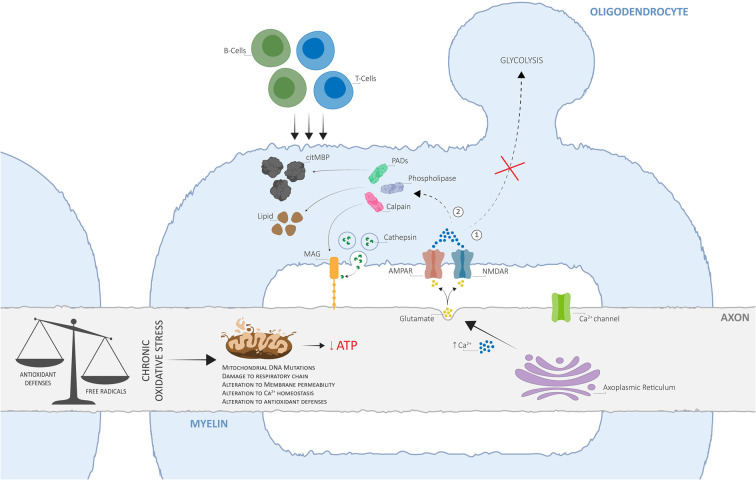
The alternative view of Multiple Sclerosis (MS) pathogenesis is based on dysfunctional axo-myelinic neurotransmission due to oxidative stress (OS)-derived mitochondrial impairment. Chronic OS mechanisms, originating from excess free radical production (due to cell metabolism, physiological stress, iron overload, environmental toxins, cigarette smoking, et cetera) in relation to the cell’s antioxidant defenses, can result in the inability of the axonal mitochondria to synthesize ATP, leading to a state of chronic virtual hypoxia (energy failure). As a result, the failure of ion transporters and the consequent influx of Na^+^ ions, activate voltage-gated Ca^2+^ channels and induce the excessive intra-axonal release of Ca^2+^. The latter stimulates the excessive vesicular release of glutamate into the periaxonal space, over-activating myelinic AMPA and NMDA receptors and resulting in excessive Ca^2+^ influx into the myelin cytoplasm, leading to two crucial pathological outcomes: **(1)** the inability of the oligodendrocyte to generate pyruvate and lactate, by glycolysis, for metabolic support to the axon; **(2)** activation of Ca^2+^-dependent calpains, phospholipases, and PADs, resulting in the degradation of myelin proteins, including myelin-associated glycoprotein (MAG), phospholipids, and in the conversion of MBP into citMBP, respectively, thus inducing focal disruptions to the myelin sheath and destabilization of the axo-myelinic synapse (AMS). The consequent release of antigenic citMBP and lipid debris can result in an adaptive immune response, driven by T- and B-cells, which can cause further inflammatory reactions. Adapted from Micu et al. (2018), with permission from the authors and SpringerNature.

Here, we propose an alternative view of MS pathogenesis, whereby OS-derived mitochondrial impairment acts as the key initiator of a disrupted axo-myelinic relationship and consequently drives the onset of primary cytodegenerative and secondary inflammatory processes (Figure 2).

Dysfunctional activation of axo-myelinic neurotransmission may be driven by complementary processes originating from excess free radical production. OS can directly affect mitochondrial functioning *via* different pathways, each leading to a state of energy failure within the affected axons (Guo et al., 2013). The presence of excess ROS, including hydrogen peroxide, puts pressure on cellular antioxidant defense systems, such as glutathione peroxidase (GPx), to restore metabolic balance (Carvalho et al., 2014). GPx is an intracellular antioxidant enzyme that reduces hydrogen peroxide to water to both limit its harmful effects and, indirectly, to modulate mitochondrial oxidative phosphorylation (Lubos et al., 2011). Consequently, with increasing levels of oxyradicals, the production of ATP by axonal mitochondria can be depleted *via* the GPx system. Additionally, the accumulation of calcium ions derived from OS processes would result in detrimental effects not only on the axon itself, *via* over-activation of Ca^2+^-dependent axonal enzymes, but also on the overlying myelin sheath, by stimulating excessive vesicular glutamate release into the periaxonal space. Elevated Ca^2+^ levels also inhibit axonal transport of mitochondria, thus immobilizing stationary mitochondria to the affected sites, leading to the degeneration of the entire axon and, consequently, neuronal injury (Su et al., 2009). Most importantly, over time, excessive Ca^2+^ entry into the myelin sheath, together with a state of energy deprivation, will cause both the myelin and the oligodendrocyte to become unable to buffer the Ca^2+^ loads, thus hindering the transport of lactate to the axon for metabolic support (Tsutsui and Stys, 2013; Micu et al., 2018; Poerwoatmodjo et al., 2020). A recent study presented evidence for impaired glycolysis and mitochondrial respiration during T-cell activation in RRMS patients (La Rocca et al., 2017). Here, these changes were associated with a down-regulation of GLUT1 resulting from the enhanced entry of calcium ions inside the myelin sheath. Also, the inability to buffer elevated Ca^2+^ levels will likely trigger a series of enzymatic pathways leading to the degradation of myelin proteins and phospholipids and thus, to axonal demyelination (Stys, 2013; Micu et al., 2018). Finally, the loss of positive charge on citrullinated myelin basic protein (citMBP), induced by the conversion of positively charged arginine residues on MBP to citrulline by peptidyl arginine deiminases (Ca^2+^-dependent enzymes; PADs), causes focal disruption to the myelin sheath (Caprariello et al., 2018; Micu et al., 2018). The consequent release of antigenic citMBP and lipid debris can result in an adaptive immune response in a host with a reactive immune system (Micu et al., 2018). Primary injury to axonal mitochondria and subsequent demyelination may, in turn, attract leukocytes from the bloodstream, which is in line with the observation that lesions and cell infiltration tend to occur around blood vessels (Gaitán et al., 2013; Lopes Pinheiro et al., 2016). This autoimmune attack, driven by peripheral T- and B-cells, resembles the primary immune-mediated inflammatory response widely described by supporters of the *outside-in* model of MS. Therefore, although the loss of metabolic support may be the initiator of progressive degeneration in MS, it is unlikely to be the only underlying mechanism; a gain of toxic function is likely to promote further, and potentially irreversible, neurological damage (Micu et al., 2018).

Given the supportive interdependence of neuronal and glial health, pathology from a single axon may spread throughout the nervous system following both a transversal pattern, as well as a process similar to Wallerian degeneration in the peripheral nervous system (PNS; Chong et al., 2012; Singh et al., 2013; Simons et al., 2014). Both spreading modalities can ultimately reflect the pattern of pathology in MS patients, whereby both a longitudinal spread across the pyramidal tracts and a more transversal spread in white and gray matter regions of the CNS can be observed (DeLuca et al., 2004). Given the comparable metabolic rate of white and gray matter, both regions are equally highly vulnerable to interruptions of energy supply (Goldberg and Ransom, 2003). Depending on the location of the primary insult, differences in clinical outcomes, as well as the extent of neurological disability, may vary.

Although the increased energy demand of an axon may not be sufficient to trigger axonal degeneration, energy deprivation likely renders neurons more vulnerable to stress (Simons et al., 2014). Moreover, age-related iron accumulation in the human brain, largely stored within the myelin sheaths, may further amplify oxidative injury to the axo-myelinic unit when it is liberated upon demyelination and may be partly responsible for the presence of activated and reactive microglia and macrophages in MS lesions (Lassmann and van Horssen, 2011; Haider, 2015). Given that processes of cytodegeneration, including early mitochondrial dysfunction associated with OS, occur during the first stages of MS, even when no apparent clinical symptom is visible, it is fair to assume that cellular antioxidant defense mechanisms may be capable of controlling the extent of oxidative damage (Fischer et al., 2012). Additionally, efficient remyelinating mechanisms may be able to sustain the effects of the oxidative injury on axonal degeneration in the early stages of the disease (Franklin and ffrench-Constant, 2008). The severity of the imbalance between OS and antioxidant defenses may contribute to the severity of MS pathology and ultimately, to neurological disability.

## Discussion

To elucidate whether cytodegeneration or an autoimmune attack may represent the initial trigger of MS, we presented an alternative view on MS pathogenesis, which identifies chronic OS-derived mitochondrial dysfunction as the main initiator of a primary cytodegeneration and secondary inflammatory response. Here, we hypothesized that mitochondrial-derived energy failure caused by oxidative injury represents a potential mechanism by which the AMS might contribute to MS pathogenesis. The mitochondria can be thought of as a “car engine,” which burns fuel and recovers the energy to drive cellular processes. When dysfunctional, however, the engine not only will not be as efficient in producing energy, but it will also create toxic by-products as a result of defective combustion. This toxic debris, generated by the degradation of myelin components as well as by the release of biochemical elements from the dysfunctional mitochondria, may elicit a secondary, inflammatory response, generating a cascade of detrimental effects that will result in severe neurological and cognitive dysfunction in MS patients.

The still hypothetical model for MS pathogenesis described here is likely describing part of the *inside-out* process and, most importantly, it does not reject the argument for the presence of immune-mediated neuroinflammation in MS. Instead, it aims to elucidate a novel mechanism that may be implicated in driving the onset of the disease. If the proposed model were to reflect the driving mechanisms of progressive degeneration and perhaps, the causative initiator of MS, antioxidant therapies could provide novel therapeutic interventions for MS patients (Adamczyk and Adamczyk-Sowa, 2016). Over-time assessment of metabolite levels of both aerobic and glycolytic energy production in patients with RRMS and PPMS may help to resolve the role that mitochondrial dysfunction and OS play in MS. Upcoming cell-based approaches for MS should also take into consideration the crucial role that a dysfunctional and highly stressed environment may play in the mechanisms of tissue repair (Witherick et al., 2010). Hence, the direct targeting of OS processes and mitochondrial dysfunction may provide a more suitable microenvironment for successful stem cell transplantations or remyelination therapies.

## Author Contributions

AL and GS conceived the study. TB performed the literature research. GS and AL provided critical revision to the manuscript. TB wrote the manuscript with input from all other authors. All authors contributed to the article and approved the submitted version.

## Conflict of Interest

The authors declare that the research was conducted in the absence of any commercial or financial relationships that could be construed as a potential conflict of interest.
